# Management of Instability following Pyogenic Sacroiliitis: Technical Case Report

**DOI:** 10.1155/2020/3409306

**Published:** 2020-02-29

**Authors:** C. Passaplan, A. Simonin, G. Maestretti, E. Gautier

**Affiliations:** Orthopaedic Surgery, Cantonal Hospital of Fribourg (HFR), Chemin des Pensionnats 2, 1700 Fribourg, Switzerland

## Abstract

**Background:**

Septic arthritis of the sacroiliac joint (SI-joint) is a rare and often delayed diagnosis. Management usually consists of intravenous antibiotics and debridement of infected tissue. However, very few reports consider the management of the secondary instability of the sacroiliac joint. *Case Presentation*. We report a case of a 16-year-old girl diagnosed with *S. aureus* pyogenic sacroiliitis who benefited from aggressive surgical debridement and primary arthrodesis for infection-related SI-joint instability in the acute infection phase.

**Conclusion:**

Diagnosis of pyogenic sacroiliitis is often delayed. Destruction of the joint can lead to chronic pain and instability. In cases of obvious intraoperative instability, primary arthrodesis could be considered in young patients.

## 1. Introduction

Infectious sacroiliitis represents less than 2% of septic arthritis [1–3]. Diagnosis is often delayed because of nonspecific clinical findings. *S. aureus* is the most common causative pathogen. Management usually consists of intravenous antibiotics. SI-joint aspiration may be challenging. However, it is recommended, if blood cultures remain sterile. Surgical debridement of infected tissue is mandatory in cases of unfavorable evolution despite parenteral antibiotics. Only few authors have addressed the definitive management of the SI-joint instability secondary to infection in the chronic phase (more than one month after onset of clinical symptoms). There is no clear consensus in the literature regarding the technique of SI-joint arthrodesis in patients with chronic infection. Different techniques using transarticular screws, anterior plate fixation, bone graft, or cage interposition are reported to perform SI-joint arthrodesis [4, 5].

## 2. Case Report

A 16-year-old girl with no medical history presented to the primary care physician with fever and low back pain radiating to the left buttock. There was no history of trauma. A viral coxitis was diagnosed, and the patient was sent home with NSAIDs and bed rest. Because of progressing pain and inability to walk, the patient presented in the emergency room 5 days later. Upon examination, she was febrile, and palpation of the SI-joint and coccyx was painful. Neurological examination was normal. Laboratory findings showed increased C-reactive protein (318 mg/l) and leucocytosis (14.2 G/l).

**Figure 1:**
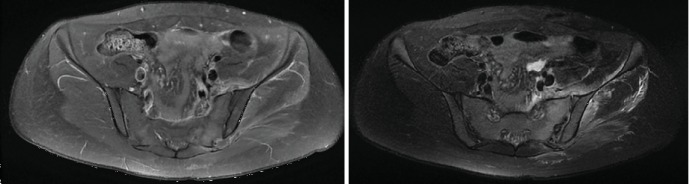
MRI of the pelvis: T1-enhanced and T2 axial views showing signal abnormality of the left sacroiliac joint, predominant in the sacral wing, characterized by hyperintensity on T2. There are significant signal abnormalities and gadolinium uptake of the musculature adjacent to the left sacroiliac joint. Anterior effusion of the left sacroiliac joint is visible with additional bulging of the posterior articular capsule.

**Figure 2:**
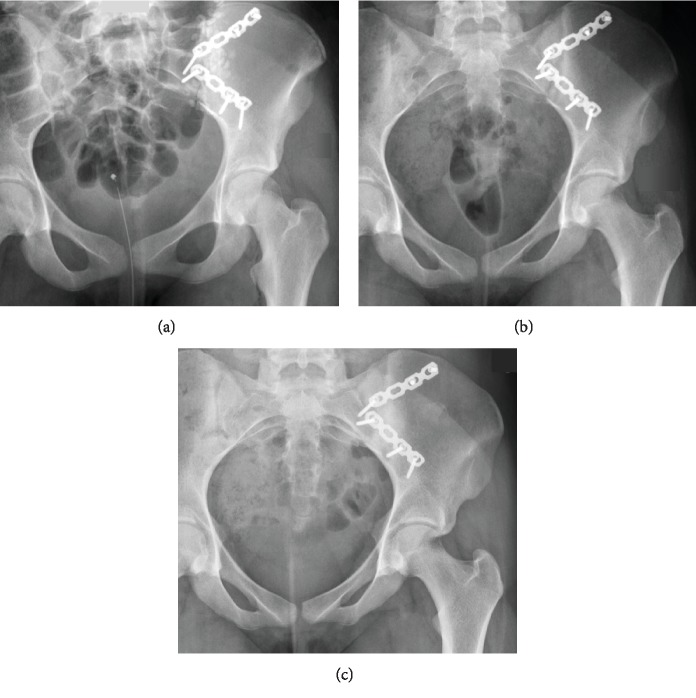
Postoperative anteroposterior pelvic radiograph showing the SI-joint arthrodesis using two plates with radiographically visible Vancomycin-loaded calcium sulphate pellets (a). Three months after surgery (b), the calcium sulphate pellets are totally resorbed. There are no signs of secondary displacement of the SI-joint, implant loosening, or failure. One year after surgery (c), the SI-joint is completely fused.

MRI of the pelvis and SI-joints showed a bulge in the intra-articular capsule of the left SI-joint and bone remodelling (Figure 1). Blood cultures were sampled, and ultrasound-guided aspiration was performed. Direct examination revealed gram-positive cocci. Intravenous antibiotics (daptomycin 350 mg/day) were started following the aspiration. Cultures became positive for *S. aureus*, and antibiotic treatment was switched to gentamicin. Because of aortic murmur, transthoracic echocardiogram was performed to rule out endocarditis. Antibiotherapy was subsequently switched to flucloxacillin. Five days after initial treatment, because of increasing fever and inflammation markers, a new MRI was performed, showing abscesses anterior to the left SI-joint. Surgical debridement was performed, using the Olerud approach to the SI-joint. After aggressive debridement and evacuation of pus, there was obvious instability of the SI-joint in the lateral-medial and craniocaudal directions. Therefore, even in the absence of significant joint destruction in the preoperative MRI, an arthrodesis was performed using two titanium reconstruction plates angulated with each other. In addition, Vancomycin-loaded calcium sulphate (Osteoset™ resorbable beat) pellets were added inside and around the articulation to provide a high local concentration of Vancomycin as described before [6] (Figure 2(a)).

**Figure 3:**
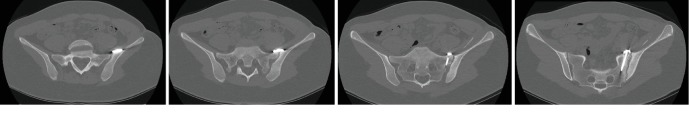
Series of axial CT scan showing early signs of fusion of the SI-joint without osteolysis of the adjacent bone.

Four days later, because of recurring pain, a new MRI was performed, showing a surgical site hematoma, which was evacuated. Subsequent clinical course was favorable; the patient was discharged at day 26 with some residual pain, normalized inflammation markers of infection, and 15 kg partial weight-bearing on the operated leg. After 6 weeks, she did not have any episode of fever and only little pain on palpation of her left SI-joint. Intravenous antibiotics were given for 9 weeks in our outpatient department, and 30 kg partial weight-bearing on the operated leg was allowed for 6 more weeks. Biological inflammatory markers were normal, and an anteroposterior pelvic radiograph was satisfactory after 3 months (Figure 2(b)). After 4 months, the patient was pain-free and could perform her usual activities without any restriction. Before allowing heavier sports activities, a CT scan was performed at 4 months revealing the first signs of joint fusion without osteolysis or secondary displacement (Figure 3). One year after surgery (Figure 2(c)), the SI-joint was no more visible on the pelvic radiograph as a sign of complete SI-joint fusion.

## 3. Discussion

Pelvic ring stability relies on osseoligamentous structures. Instability is mainly caused by traumatic injuries. However, chronic conditions like infectious or inflammatory diseases may also affect the integrity of the sacroiliac joint [2]. Pyogenic sacroiliitis is rare and diagnosis is often delayed. However, it is usually not associated with instability when appropriate treatment is conducted before destruction of the SI-joint. Management usually consists of surgical debridement and several weeks of intravenous antibiotherapy. However, very few reports consider the infection-related SI-joint instability. Many authors recommend 2-3 months of non-weight-bearing [2]. Long-term outcome is lacking in most of the series and case reports [7–21]. Chronic SI-joint pain is reported in a high proportion of cases (33% to 43.5%) [3, 22, 23]. The cause of the pain has not been investigated in retrospective studies. But chronic SI-joint instability could be a causative phenomenon.

Arthrodesis is normally reserved as a salvage procedure in case of ineffective conservative therapy or by severity criteria as septicaemia or neurological deficits in the acute phase or secondary instability due to joint destruction in the chronic phase of the infection. The most appropriate surgical regime is still discussed controversially in the literature: open procedure for extensive debridement and fusion of the joint are described in some series as a current approach [1, 2, 22]. The surgical approach can be posterior, anterior, or mixed, depending on the location of the abscess on the MRI. Schubert et al. [24] described the first series of primary arthrodesis with autologous bone graft with a posterior approach of the sacroiliac joint in case of neurological deficits, abscess formation, or septicaemia, with good clinical results. Ahmed et al. [4] described a series of eleven cases of debridement combined to arthrodesis performed with a variety of surgical techniques (posterior or combined approaches, bone graft with/without cage and/or screws) and recommended a joint fusion in chronic cases or in patients in bad general condition even with moderate destruction of the sacroiliac joint. Recently, some cases of minimal invasive sacroiliac joint fusion were recently described in the literature showing good results in case of chronic sacroiliac dysfunction. Anton et al. [25] described a pyogenic sacroiliitis treated with minimally invasive joint fusion (SI Fusion System Medtronic) with a good long-term outcome. Wandermann et al. [26] reported a case of chronic infectious sacroiliitis treated with a combination of joint debridement, biologic fusion with bone graft, and nonbiologic functional fusion using titanium ingrowth rods, all performed in a minimally invasive fashion with good results at 2 years of follow-up. Wise and Dall [27] described a series of patients presenting after failed conservative therapy of pyogenic sacroiliitis. Fusion was achieved by percutaneous insertion of cages on the posterior side of the SI-joint.

In cases presenting a destruction of the SI-joint or obvious intraoperative instability, like in our case, primary arthrodesis could be considered, in order to prevent chronic pain and instability. Minimally invasive techniques are described for the treatment of sacroiliac joint infection showing good results, low complication rates, and short recovery. Nevertheless, in case of pyogenic SI-joint infection, an open procedure is a successful alternative. The anterior approach to the SI-joint allows debridement of all infected tissue, complete removal of the infected cartilage by distraction of the SI-joint, local administration of antibiotics using Vancomycin-loaded calcium sulphate pellets, and stable fixation by means of two reconstruction plates.

## 4. Conclusion

Diagnosis of pyogenic sacroiliitis is often delayed. The infectious process can lead to the destruction of the SI-joint with chronic pain due to persistent infection and instability. In cases with destruction of the SI-joint and obvious intraoperative instability, primary arthrodesis could be considered even in the acute phase of the infection in order to prevent chronic pain and instability. Our case is to our knowledge the first report of a one-stage debridement and primary plate fixation of an acute pyogenic SI-joint infection.

## References

[1] Schaad U. B., McCracken GH Jr, Nelson J. D. (1980). Pyogenic arthritis of the sacroiliac joint in pediatric patients. *Pediatrics*.

[2] Kanakaris N. K., Psarakis S., Chalidis B., Kontakis G., Giannoudis P. V. (2009). Management of pelvic instability secondary to chronic pyogenic sacroiliitis: case report. *Surgical Infections*.

[3] Horai Y., Izumikawa K., Oka S. (2014). Methicillin-resistant Staphylococcus aureus-related septic pulmonary embolism and sacroiliitis treated with long-term linezolid in a patient with adult-onset Still’s disease. *Internal Medicine*.

[4] Ahmed H., Siam A. E., Gouda-Mohamed G. M., Boehm H. (2013). Surgical treatment of sacroiliac joint infection. *Journal of Orthopaedics and Traumatology*.

[5] Spiker W. R., Lawrence B. D., Raich A. L., Skelly A. C., Brodke D. S. (2012). Surgical versus injection treatment for injection-confirmed chronic sacroiliac joint pain. *Evidence-Based Spine-Care Journal*.

[6] Wahl P., Guidi M., Benninger E. (2017). The levels of vancomycin in the blood and the wound after the local treatment of bone and soft-tissue infection with antibiotic-loaded calcium sulphate as carrier material. *Bone & Joint Journal*.

[7] Donzelli A., Samara E., Spyropoulou V., Juchler C., Ceroni D. (2017). Pediatric sacroiliitis: clinical and microbiologic differences between infants and children-adolescents. *The Pediatric Infectious Disease Journal*.

[8] Ghedira Besbes L., Haddad S., Abid A., Ben Meriem C., Gueddiche M. N. (2012). Pyogenic sacroiliitis in children: two case reports. *Case Reports in Medicine*.

[9] Kim S., Lee K. L., Baek H. L., Jang S. J., Moon S. M., Cho Y. K. (2013). A case of acute pyogenic sacroiliitis and bacteremia caused by community-acquired methicillin-resistant Staphylococcus aureus. *Infect Chemother*.

[10] Shemer A., Eshed I., Levinkopf M. (2018). Septic sacroiliitis: a diagnostic challenge for the clinician. *The Israel Medical Association Journal*.

[11] Hermet M., Minichiello E., Flipo R. M. (2012). Infectious sacroiliitis: a retrospective, multicentre study of 39 adults. *BMC Infectious Diseases*.

[12] Jayamali W. D., Herath H. M. M. T. B., Kulatunga A. (2017). A young female presenting with unilateral sacroiliitis following dengue virus infection: a case report. *Journal of Medical Case Reports*.

[13] Antonelli M. J., Magrey M. (2017). Sacroiliitis mimics: a case report and review of the literature. *BMC Musculoskeletal Disorders*.

[14] Doita M., Yoshiya S., Nabeshima Y. (2003). Acute pyogenic sacroiliitis without predisposing conditions. *Spine*.

[15] Zejden A., Jurik A. G. (2017). Anatomy of the sacroiliac joints in children and adolescents by computed tomography. *Pediatric Rheumatology Online Journal*.

[16] McHugh R. C., Tiede J. M., Weingarten T. N. (2008). Clostridial sacroiliitis in a patient with fecal incontinence: a case report and review of the literature. *Pain Physician*.

[17] Bellazreg F., Alaya Z., Hattab Z. (2016). Infectious sacroiliitis in tunisian centre: retrospective study of 25 cases. *The Pan African Medical Journal*.

[18] Aydin F., Özçakar Z. B., Çakar N. (2019). Sacroiliitis in children with familial mediterranean fever. *Journal of Clinical Rheumatology*.

[19] Almoujahed M. O., Khatib R., Baran J. (2003). Pregnancy-associated pyogenic sacroiliitis: case report and review. *Infectious Diseases in Obstetrics and Gynecology*.

[20] Woytala P. J., Sebastian A., Błach K., Silicki J., Wiland P. (2018). Septic arthritis of the sacroiliac joint. *Reumatologia*.

[21] Elsammak M., Hanna H., Ghazal A., Edeen F. B., Kandil M. (2006). Diagnostic value of serum procalcitonin and C-reactive protein in Egyptian children with streptococcal tonsillopharyngitis. *The Pediatric Infectious Disease Journal*.

[22] Leroux J., Bernardini I., Grynberg L. (2015). Pyogenic sacroiliitis in a 13-month-old child: a case report and literature review. *Medicine*.

[23] Ennis H. E., Ialenti M. N., Jose J., Baraga M. (2017). A rare case of isolated salmonella species group B sacroiliitis in a healthy collegiate-level swimmer: a case report. *JBJS Case Connector*.

[24] Schubert T., Bruns J., Dahmen G. (1993). Results of surgical therapy of bacterial sacroiliitis with primary arthrodesis. *Langenbecks Archiv für Chirurgie*.

[25] Anton G., Tong D., Little T., Soo T. M. (2019). Minimally invasive sacroiliac joint fusion for the treatment of brucella pyogenic sacroiliitis: a case report. *Cureus*.

[26] Wanderman N., Thurn J., Wyffels M., Sembrano J. N. (2016). Successful treatment of *Mycobacterium gordonae* sacroiliitis using a novel minimally invasive sacroiliac joint arthrodesis: A case report. *JBJS Case Connector*.

[27] Wise C. L., Dall B. E. (2008). Minimally invasive sacroiliac arthrodesis: outcomes of a new technique. *Journal of Spinal Disorders & Techniques*.

